# Whole-genome resequencing of Xishuangbanna fighting chicken to identify signatures of selection

**DOI:** 10.1186/s12711-016-0239-4

**Published:** 2016-08-26

**Authors:** Xing Guo, Qi Fang, Chendong Ma, Bangyuan Zhou, Yi Wan, Runshen Jiang

**Affiliations:** College of Animal Science and Technology, Anhui Agricultural University, Hefei, 230036 People’s Republic of China

## Abstract

**Background:**

Selective breeding for genetic improvement is expected to leave distinctive selection signatures within genomes. The identification of selection signatures can help to elucidate the mechanisms of selection and accelerate genetic improvement. Fighting chickens have undergone extensive artificial selection, resulting in modifications to their morphology, physiology and behavior compared to wild species. Comparing the genomes of fighting chickens and wild species offers a unique opportunity for identifying signatures of artificial selection.

**Results:**

We identified selection signals in 100-kb windows sliding in 10-kb steps by using two approaches: the pooled heterozygosity $({\text{H}}_{\text{p}} )$ and the fixation index $(F_{\text{ST}} )$ between Xishuangbanna fighting chicken (YNLC) and Red Jungle Fowl. A total of 413 candidate genes were found to be putatively under selection in YNLC. These genes were related to traits such as growth, disease resistance, aggressive behavior and energy metabolism, as well as the morphogenesis and homeostasis of many tissues and organs.

**Conclusions:**

This study reveals mechanisms and targets of artificial selection, which will contribute to improve our knowledge about the evolution of fighting chickens and facilitate future quantitative trait loci mapping.

**Electronic supplementary material:**

The online version of this article (doi:10.1186/s12711-016-0239-4) contains supplementary material, which is available to authorized users.

## Background

Domesticated chickens have long been bred for entertainment and consumption [1]. Fighting chickens are a group of ancient breeds that have been bred for the purpose of cock fighting and have played an important role in the development of human culture [2]. The Xishuangbanna fighting chicken (YNLC) is a typical fighting chicken breed that has been subjected to strong artificial selection, which has led to remarkable phenotypic characteristics in morphology, physiology, and behavior. The YNLC represents an excellent model that can provide new insights into the influence of artificial selection on genetic variation and how this shapes phenotypic diversity.

Selection leads to specific changes in the patterns of variation among selected loci and in neutral loci linked to them. These genomic footprints of selection are known as selection signatures and can be used to identify loci that have been subjected to selection [3]. Various statistical approaches have been proposed for the detection of selection signatures [4–7]. The pooled heterozygosity $( {\text{H}}_{\text{p}} )$ statistic is a variability estimator based on allele counts across sliding windows of adjacent loci and can be used to identify regions that deviate from the norm [8]. The fixation index $(F_{\text{ST}} )$ can be used to quantify the degree of genetic differentiation among populations based on differences in allele frequencies [9]. Both ${\text{H}}_{\text{p}}$ and $F_{\text{ST}}$ statistics are useful for the detection of selection signatures [10]. In this study, we used two outlier approaches (${\text{H}}_{\text{p}}$ and $F_{\text{ST}}$) to detect signatures of selection in YNLC and provide insights into the mechanisms of evolution of this specific breed.

## Methods

### Re-sequencing of chicken samples, mapping and SNP calling

We downloaded the genomic data for eight YNLC and six wild Red Jungle Fowl (RJF) from the EMBL-EBI database (see Additional file 1: Table S1). Details about the sequenced samples and method of sequencing are in [11, 12]. As mentioned in these two papers, individual DNA libraries with an insert size of 500 bp were constructed and sequenced by using the Illumina HiSeq 2000 platform. The samples were sequenced at a genome coverage of 11.1X to 36.6X (see Additional file 1: Table S1) which is appropriate for analysis of selective sweeps [13, 14]. All reads were preprocessed for quality control and filtered using our in-house script in Perl. Before aligning reads onto the reference genome, we performed the following quality checks [15]:

1. If there were more than 10 % unidentified nucleotides (N) or 10-nucleotide adaptors (<10 % mismatch) in either of the paired reads, the reads were removed.
2. If there were more than 50 % low-quality bases (Q ≤ 5) in either of the paired reads, the reads were removed.
3. Duplicated reads were also removed, only paired-end reads were kept for subsequent analyses.

High-quality paired-end reads were mapped to the chicken reference genome sequence (ftp://ensembl.org/pub/release-67/fasta/gallus_gallus/dna/) using the BWA software [16] and the command ‘mem -t 4 -k 32 -M’. Duplicated reads were removed using the picard package [16]. After alignment, we performed single nucleotide polymorphism (SNP) calling on a population scale for the two groups (YNLC and RJF) using SAMtools [17]. The ‘mpileup’ command was used to identify SNPs with the parameters ‘-m 2 -F 0.002 -d 1000’. Putative functional effects of SNPs were annotated using the ANNOVAR package [18]. To exclude SNP calling errors caused by incorrect mapping, only high-quality SNPs (root-mean-square mapping quality ≥20, coverage depth ≥4 and ≤1000, distance between adjacent SNPs ≥5 bp and rate of missing data within each group <50 %) were retained for subsequent analyses.

### Analysis of selection signatures

We used allele frequencies at variable sites to identify signatures of selection in 100-kb windows with a step size of 10 kb by using two approaches. For each window, we calculated ${\text{H}}_{\text{p}}$ and $F_{\text{ST}} .$ At each detected SNP position, we counted the number of reads corresponding to the most and least frequently observed allele (nMAJ and nMIN, respectively) for each breed pool (i.e. all eight samples for YNLC and all six samples for RJF were combined, respectively). For each window, we calculated ${\text{H}}_{\text{p}}$ as follows [6]:

$${\text{H}}_{\text{p}} = 2\sum {\text{nMAJ}}\sum {\text{nMIN}}\left/{\left( {\sum {\text{nMAJ}}\sum {\text{nMIN}}} \right)^{2}}. \right.$$

Subsequently, individual ${\text{H}}_{\text{p}}$ values were ${\text{Z}}$-transformed as follows:

$${\text{ZH}}_{\text{p}} = {\left( {{\text{ZH}}_{\text{p}} - {\upmu\text{ZH}}_{\text{p}} } \right)} \left/{{\sigma\text{ZH}}_{\text{p}}}\right. .$$

$F_{\text{ST}}$ was calculated from the allele frequencies (not the allele counts) using the standard equation according to the principles of population genetics [19]:

$$F_{\text{ST}} = {{\text{P}}_{\text{i}} \_{\text{total}} - {\text{P}}_{\text{i}} \_{\text{within}}} \left/{{\text{P}}_{\text{i}} \_{\text{within}}},\right.$$

where ${\text{P}}_{\text{i}} \_{\text{within}} = \left( {{\text{P}}_{\text{i}} \_{\text{population}}1 + {\text{P}}_{\text{i}} \_{\text{population}}2} \right)/2,$ and ${\text{P}}_{\text{i}} = 1 - {\text{fA}}^{2} - {\text{fT}}^{2} - {\text{fC}}^{2} - {\text{fG}}^{2}$ with ${\text{fN}}$ being the frequency of nucleotide ${\text{N }}\left( {{\text{i}}.{\text{e}}. {\text{A}}, {\text{T}}, {\text{C or G}}} \right),\,{\text{P}}_{\text{i}} \_{\text{total}}$ is the total ${\text{P}}_{\text{i}}$ for which allele frequencies in both populations are averages and ${\text{P}}_{\text{i}}$ is calculated as above.

The $F_{\text{ST}}$ values were Z-transformed as follows:

$${\text{Z}}\left( {F_{\text{ST}} } \right) = {\left( {F_{\text{ST}} - {\upmu }F_{\text{ST}} } \right)}\left/{\sigma F_{\text{ST}}},\right.$$

where ${\upmu }F_{\text{ST}}$ is the mean $F_{\text{ST}} ,$ and $\sigma F_{\text{ST}}$ is the standard deviation of $F_{\text{ST}}$ [20]. ${\text{H}}_{\text{p}}$ and $F_{\text{ST}}$ were calculated by using our in-house script in Perl. The major challenge of such analyses is to exclude signals caused by demographic events and population structure. It is difficult to assign strict thresholds to distinguish selection and drift. We surveyed published literature and used an empirical procedure according to previous studies [21, 22]. Putatively selected regions were located in fully overlapping windows with an extremely low ${\text{ZH}}_{\text{p}}$ value (top 5 % level) and extremely high ${\text{Z}}F_{\text{ST}}$ values (top 5 % level).

### Functional enrichment analysis

The genes putatively under selection were submitted to g:profiler (http://biit.cs.ut.ee/gprofiler/. Version: r1488_e83_eg30.) for enrichment analysis of the Gene Ontology (GO) and KEGG pathways. All chicken genes that are annotated in Ensembl were used as the background set. Benjamini–Hochberg FDR (false discovery rate) was used for correcting the P values. Only terms with a P value <0.05 were considered as significant and listed.

## Results

### Detection of SNPs

A total of 16.40 × 10^6^ SNPs were identified from the genomes of 14 individuals, i.e. eight YNLC (13.15 × 10^6^ SNPs) and six RJF individuals (13.87 × 10^6^ SNPs) (see Additional file 2: Table S2). Most SNPs identified for the YNLC individuals were located in intergenic and intron regions (57.16 and 38.77 %, respectively); 1.36 % of these 13.15 × 10^6^ SNPs were predicted to be within protein-coding regions and 0.40 % as amino acid altering mutations (non-synonymous and stop gain/loss); 1.30 % of the 13.15 × 10^6^ SNPs were within 1-kb regions upstream or downstream of the transcription start or end sites, and thus may have a possible role in transcriptional regulation, with 510 SNPs of these residing within splice sites (Table 1; see Additional file 2: Table S2).

**Table 1:** Summary and annotation of SNPs in YNLC^a^

Category^b^	YNLC
Upstream	186,263
Exonic	
Stop gain	379
Stop loss	51
Synonymous	126,014
Non-synonymous	51,933
Intronic	5,098,263
Splicing	510
Downstream	163,867
Upstream/downstream	6747
Intergenic	7,515,849

^a^YNLC: Xishuangbanna fighting chicken

^b^Upstream: a variant that is located in the 1-kb region upstream of the gene start site; stop gain: a non-synonymous (ns) SNP that leads to the creation of a stop codon at the variant site; stop loss: a non-synonymous SNP that leads to the elimination of a stop codon at the variant site; splicing: a variant within 2 bp of a splice junction; downstream: a variant that is located in the 1-kb region downstream of the gene end site; upstream/downstream: a variant that is located in the downstream and upstream regions of two genes

### Genome-wide selective sweep signals

**Fig. 1:**
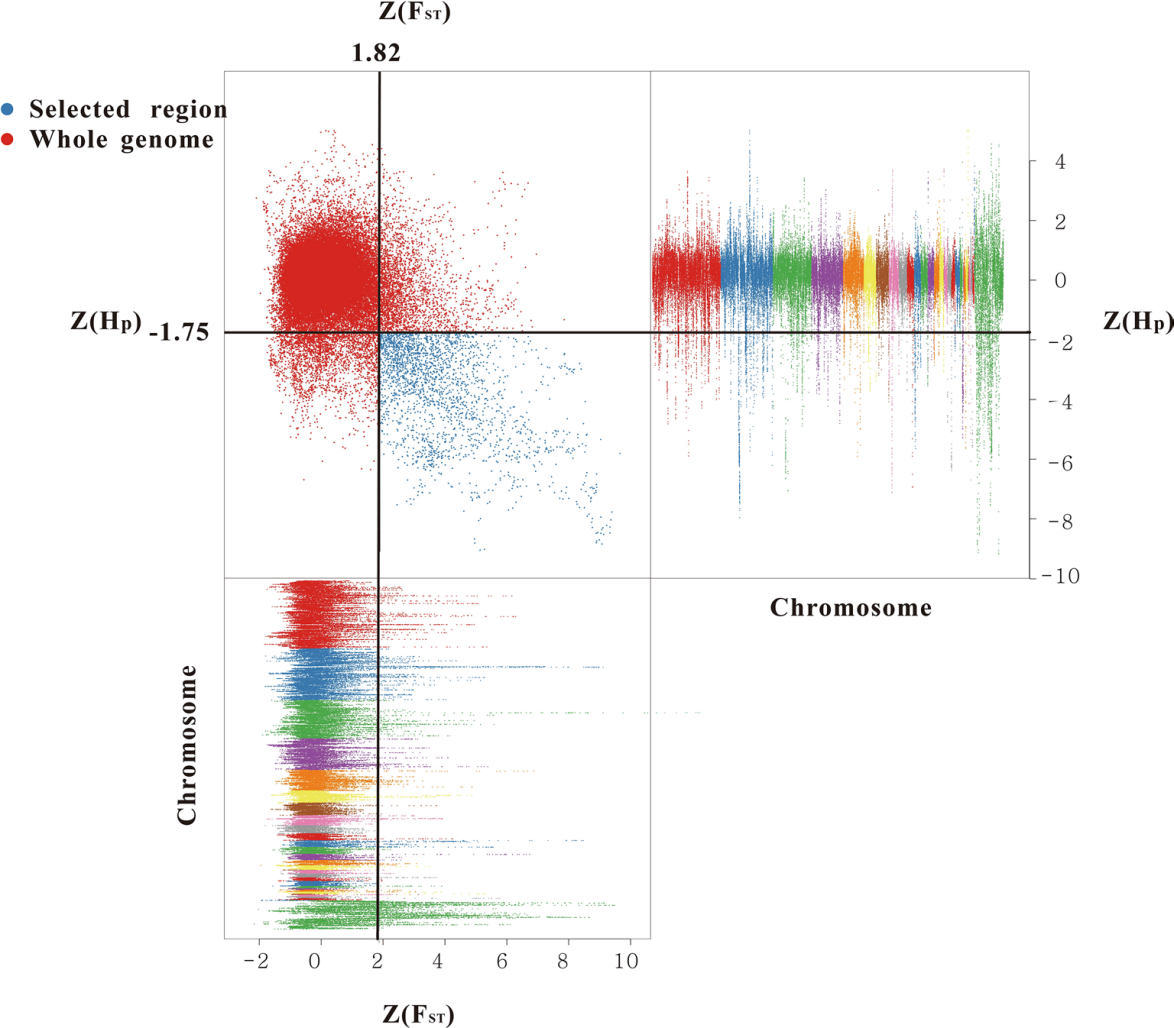
Distribution of ${\text{ZH}}_{\text{p}}$ and ${\text{Z}}F_{\text{ST}}$ calculated for 100-kb windows sliding in 10-kb steps. *Blue points* identify Xishuangbanna game chicken (YNLC) genomic regions with both an extremely low ${\text{ZH}}_{\text{p}}$ value (top 5 % level) and an extremely high ${\text{Z}}F_{\text{ST}}$ value (top 5 % level)

To detect selection signatures, we searched the genome of the YNLC chicken for regions with reduced ${\text{H}}_{\text{p}}$ and increased genetic distances to the RJF genome $(F_{\text{ST}} ).$ Putatively selected genes were located by extracting windows that simultaneously presented extremely low ${\text{ZH}}_{\text{p}}$ (top 5 % level, ${\text{ZH}}_{\text{p}} = - 1.75$) and extremely high ${\text{Z}}F_{\text{ST}}$ (top 5 % level, ${\text{Z}}F_{\text{ST}} = 1.82$). A total of 413 candidate genes (Fig. 1 , Additional file 3: Table S3, Additional file 4: Figure S1) were identified in the YNLC genome, which should harbor genes that underwent selection for fighting aptitude. We compared our results with those previously reported by Rubin et al. [6] and detected 91 overlapping genes between the two studies (see Additional file 3: Table S3). We searched for significantly overrepresented GO terms and KEGG pathways among the candidate genes that are specific to YNLC. The most enriched clusters were related to immunity, disease resistance, organ development, response to stimulus, and metabolic processes (see Additional file 5: Table S4, Additional file 6: Table S5).

## Discussion

Four hundred and thirteen genes were discovered in our study, of which 91 overlapped with those reported in [6] and among these, we identified several notable domestication-related genes i.e.: *IGF1* (*insulin*-*like growth factor 1*), which encodes a peptide that has a similar molecular structure to that of insulin and is a candidate gene for avian growth [6]; *BCO2* (*β*-*carotene oxygenase 2*), which is associated with yellow skin in domestic chickens [23]; and *NELL1* (*NEL*-*like 1*) which is assumed to be related to skeletal integrity in chickens [24]. Positive selection on these genes in the YNLC was expected since domestic chickens collectively share morphology and physiology shifts that accompanied domestication [25].

Many genes that were putatively under selection and identified in our study were not reported by Rubin et al. [6]. Functional enrichment analysis of genes that are specific to the YNLC breed revealed that many candidate genes are related to immunity and disease resistance (see Additional file 5: Table S4), which may reflect artificial selection for individuals with improved innate immunity and disease resistance.

Among the identified candidate genes, quite a few are involved in organ development (see Additional file 5: Table S4), e.g. *CBFB* (*core*-*binding factor subunit beta*) and *GRHL3* (*grainyhead*-*like 3*), which are critical for growth and development of the craniofacial skeleton [26, 27]. These genes may explain why fighting chickens have a wider mandibular joint and frontal bone as compared to other breeds [28]. Many of the genes identified are related to limb development, i.e. *Gli3* (*transcriptional activator Gli3*) and *PTCH1* (*patched 1*), which are involved in the hedgehog (Hh) signal transduction pathway that controls the patterning, growth, morphogenesis and homeostasis of many tissues [29], such as digit patterning [30] and limb development [31]; *EFNA5* (*ephrin*-*A5*), a GPI-anchored ephrin-Aligand that binds to the Eph receptors, is pivotal in cell migration in the avian forelimb [32]. Compared with other breeds, fighting chickens exhibit larger hindlimb and forelimb muscles, especially for triceps surae and biceps brachii [33], which may reflect adaptation to running and jumping that are essential traits in this breed. The triceps surae muscles assist in extending the foot joints, while the large triceps surae muscles allow fighting chickens to have a high level of jumping performance. The biceps brachii muscles facilitate strong flapping of the wings and act as powerful flexors of the elbow joint to support both jumping and hitting actions. In addition, fighting chickens have long legs, an extended hip joint, and a curved knee joint [33, 34], which indicate that they have adapted to running and upright posture.

**Fig. 2:**
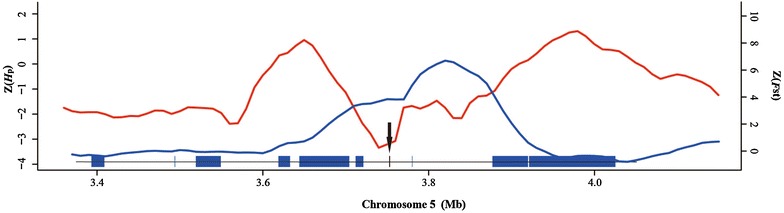
Example of the *BDNF* gene (*arrow*) with selection signals in Xishuangbanna game chicken (YNLC). ${\text{Z}}F_{\text{ST}}$ (*blue*) and ${\text{ZH}}_{\text{p}}$ (*red*)

Fighting chickens are bred specifically for cockfighting and fighting cocks possess congenital aggression towards all males of the same species. Several of the identified genes are related to aggressive behavior (Table 2). For example, the *brain*-*derived neurotrophic factor* (*BDNF*) gene (Fig. 2), a member of the nerve growth factor gene family, plays a major role in neuronal growth, proliferation, differentiation and neuronal survival [35]. A mutation in the human *BDNF* gene has been reported to be correlated with aggressive behavior in humans [36]. Furthermore, *BDNF* loss-of-function mice have been used as a model to study animal aggression [37]. Another gene *neurotensin*/*neuromedin N precursor* (*NTS*) encodes a common precursor for neurotensin (NT) and neuromedin N (NN). NT is involved in interactions with dopamine [38] and corticotropin-releasing factor (CRF) signaling [39], two neurotransmitter systems known to modulate aggressive behavior [40, 41]. Furthermore, *NT* mRNA levels were shown to be significantly reduced in high maternal aggression mice [42].

**Table 2:** Putative selected genes involved in aggressive behavior

Gene ID	Gene name	References
ENSGALG00000012163	*BDNF*	[36, 37]
ENSGALG00000027192	*NTS*	[42]
ENSGALG00000003163	*GNAO1*	[43]

Cockfighting is a very toilsome and furious form of exercise. The ability to sustain and effectively allocate fuel substrates for oxidative metabolism is critical for cockfighting. Energy metabolism-related genes were found to be under selection in YNLC (see Additional file 5: Table S4). The *RICTOR* (*RPTOR independent companion of MTOR complex 2*) gene encodes an essential subunit of the target of the rapamycin (mTOR) complex (mTORC) 2. In fat cells, RICTOR/mTORC2 plays an important role in whole-body energy homeostasis [44]. The *SDHB* (*succinate dehydrogenase* (*SDH*) *subunit B*) gene encodes a crucial metabolic enzyme that is involved in the respiratory chain and Krebs cycle [45]. Positive selection of these genes may represent adaptation of the energy metabolism in fighting chickens.

## Conclusions

In this work, we used two distinct methods to detect selection signatures across the genomes of YNLC and RJF chicken. Our analyses identified genes under positive selection in YNLC, which included genes related to aggressive behavior, immunity, energy metabolism and tissue and organ development. Our data will help improve our understanding of the mechanisms and identify the targets of artificial selection in fighting chickens and facilitate future quantitative trait loci (QTL) mapping.

## Additional files

10.1186/s12711-016-0239-4 Summary of the downloaded chicken genome re-sequencing data. Description: The table provides sequencing depth and accession numbers from the downloaded chicken genome.
10.1186/s12711-016-0239-4 Summary and annotation of SNPs in Xishuangbanna fighting chicken (YNLC) and Red Jungle Fowl (RJF).
10.1186/s12711-016-0239-4 Putatively selected genes (top 5 % level of ${\text{ZH}}_{\text{p}}$ and ${\text{Z}}F_{\text{ST}}$ values) in the Xishuangbanna fighting chicken (YNLC).
10.1186/s12711-016-0239-4 Genome-wide distribution of ${\text{ZH}}_{\text{p}}$ and ${\text{Z}}F_{\text{ST}}$ along chromosomes. This figure presents the putatively selected regions on YNLC chromosomes.
10.1186/s12711-016-0239-4 GO analysis for all genes under selection in YNLC. The table provides the significantly overrepresented gene ontology (GO) terms among putatively selected genes in YNLC.
10.1186/s12711-016-0239-4 KEGG analysis for all genes under selection in YNLC. The table provides the significantly enriched KEGG pathways among putatively selected genes in YNLC.
