# Supplementary material for: Whole-genome resequencing of Xishuangbanna fighting chicken to identify signatures of selection
Source: Genet Sel Evol. 2016 Aug 26;48(1):62. doi: 10.1186/s12711-016-0239-4 (PMC5000499; doi:10.1186/s12711-016-0239-4)
Supplement: Supplementary file 4 — 10.1186/s12711-016-0239-4 Genome-wide distribution of ${\text{ZH}}_{\text{p}}$ZHp and ${\text{Z}}F_{\text{ST}}$ZFST along chromosomes. This figure presents the putatively selected regions on YNLC chromosomes. [file 12711_2016_239_MOESM4_ESM.docx]

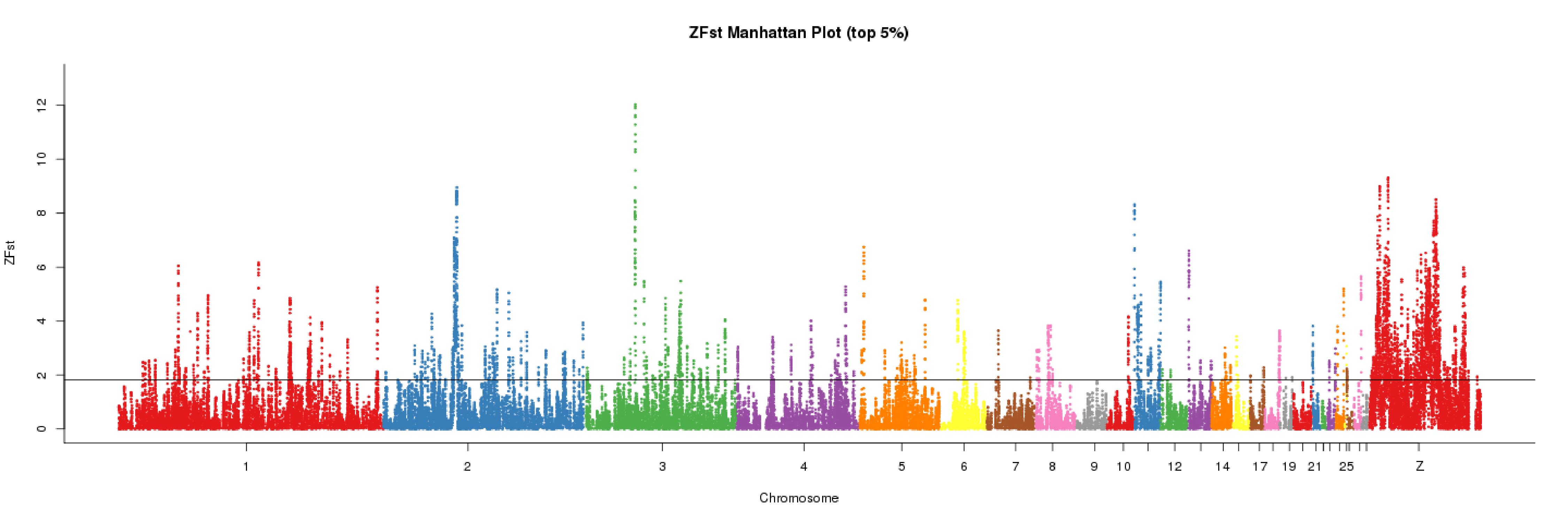

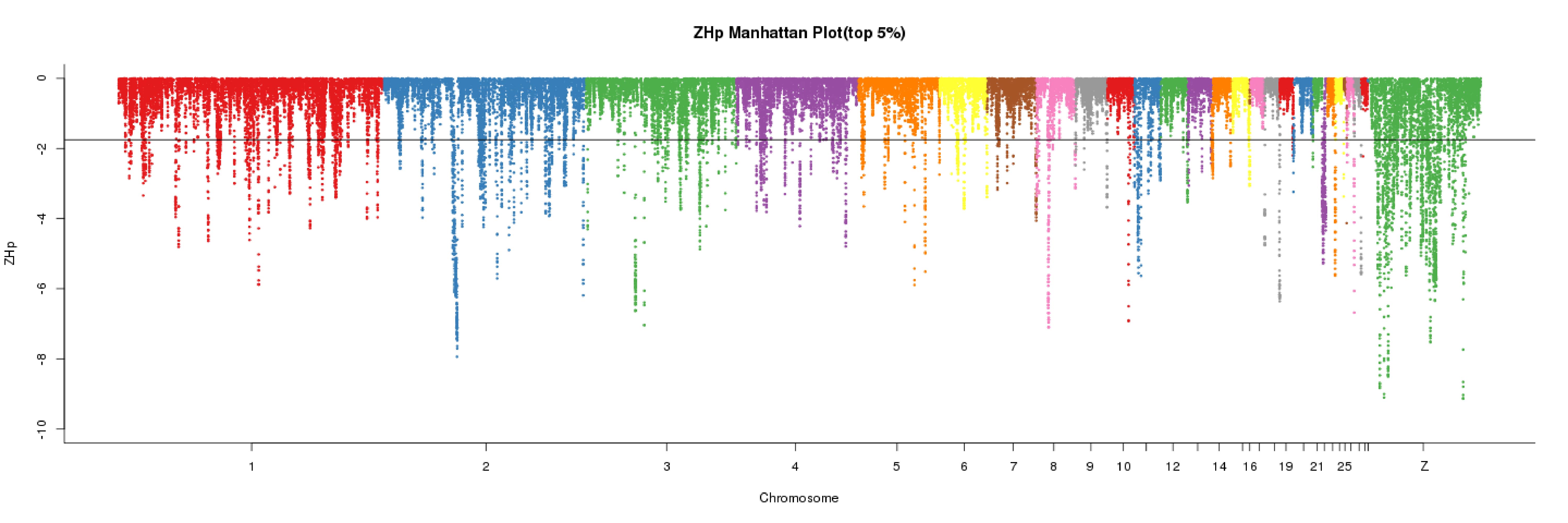


Figure S1. Genome-wide distribution of ZF_ST_ and ZH_P_ along chromosomes. A dashed horizontal line indicates the top 5% level (ZF_ST_=1.82, ZH_P_= -1.75)　used for extracting outliers
